# Assessment of Quality of Life in Bronchial Asthma Patients

**DOI:** 10.7759/cureus.10845

**Published:** 2020-10-08

**Authors:** Rashid Ali, Naseem Ahmed, Muhammad Salman, Sofia Daudpota, Madiha Masroor, Muhammad Nasir

**Affiliations:** 1 Chest Medicine, Jinnah Postgraduate Medical Centre, Karachi, PAK; 2 Paediatrics and Child Health, The Aga Khan University, Karachi, PAK; 3 Pulmonary Medicine, Jinnah Postgraduate Medical Centre, Karachi, PAK; 4 Internal Medicine, Critical Care Unit, South City Hospital, Karachi, PAK; 5 Critical Care Medicine, Anesthesiology, South City Hospital, Karachi, PAK

**Keywords:** asthma, bronchial asthma, copd, hrqol

## Abstract

Introduction

Asthma, a well-known chronic respiratory disease, is common worldwide. This study aimed to assess the quality of life in bronchial asthma patients and to determine the factors leading to poor quality of life among these patients.

Materials and methods

A cross-sectional study was conducted at a public sector hospital. The sample size was calculated as 134, with a nonprobability consecutive sampling technique. The Ethical Review Committee approved the study protocol. Demographic and asthma quality of life data were collected via a questionnaire. Data were analyzed IBM SPSS Statistics for Windows, Version 19.0 (Armonk, NY: IBM Corp.). Multivariate logistic regression was performed to observed the effect of these variables on the poor quality of life. A regression coefficient and odds ratio with a confidence interval of 95% and P-value ≤ .05 were taken as significant.

Results

The average age of patients was 40.6 ± 9.5 years. In this study, 96 of 134 patients (71.4%) with bronchial asthma reported a poor quality of life. In the univariate analysis, advanced age (≥ 40 years), obesity, being female, family history of asthma, pets at home, and moderate severity of asthma significantly contributed to poor quality of life. Multivariate logistic regression was performed, and it was observed that advanced age (≥ 40 years), being female, a pet at home, and moderate severity of asthma were four to 13 times more likely to predict a poor quality of life for patients with bronchial asthma.

Conclusions

The severity of asthma significantly contributed to poor quality of life. Health facilitators should look into the causes of such risk to increase the perception of health-related quality of life (HRQoL) among asthma patients.

## Introduction

Asthma, a well-known chronic respiratory disease, is one of the most common global problems, with an estimated total of 300 million affected individuals, comprising all age groups and exerting a significant burden on patients and their families [1]. The asthma load report by the Global Initiative for Asthma indicates that the prevalence of asthma ranges from 1% to 18% of the population [1,2]. Patients diagnosed with chronic obstructive pulmonary disease (COPD) have significantly reduced health-related quality of life (HRQoL) and account for 250,000 deaths per year worldwide [3]. HRQoL is a vital factor in pulmonary illnesses [4], and COPD can reduce HRQoL via physical and psychosocial complications [5].

Though asthma negatively impacts the quality of life of the patients, the core influencing factors are not fully understood. The most severe forms of asthma are integrated with a poor HRQoL with nonlinear coordination [6,7]. Factors need to be identified to improve HRQoL [6-8]. Motaghi-Nejad et al. found that asthma has a negative influence on the HRQoL in 48.3% of patients [9]. Gonzalez-Barcala et al. analyzed factors associated with a poor HRQoL like obesity (24.9%), being female (28%), advanced age (21.7%), low education (56.5%), family history of asthma (24.4%), moderate persistent severity of asthma (36.4%), being a smoker (23.3%), and pets at home (24.4%) [10]. HRQoL interventions are integrated with several clinical trials [11-13]. Furthermore, no local study data on effective pharmacotherapy are available. The study aims to assess the relationship between asthma severity and HRQoL and determine the primary factors in asthma that impact patient quality of life.

## Materials and methods

This cross-sectional study was held at the outpatient department of a public sector hospital. The sample size (N=134) was estimated on the prevalence rate of factors (12.7%) through the help of the World Health Organization sample size calculator [12]. A confidence interval (CI) of 95% and a non-probability consecutive sampling technique was used during data collection. All patients with mild to moderate persistent asthma were classified according to the tool defined by the Guidelines for the Diagnosis and Management of Asthma [13]. Patients ranged from 18 to 60 years in age and were of either sex. Patients had clinical stability, no exacerbation, and had asthma for at least six months and treatment for the prior two weeks. Patients unwilling to participate were excluded from the study along with patients with acute severe/persistent asthma and those with a history of severe respiratory tract infection/chronic rhino-sinusitis, pulmonary tuberculosis, or lung cancer the past month. Ethical approval was granted from the Institutional Ethical Review Committee.

All subjects fulfilling the eligibility criteria were enrolled after providing informed verbal and written consent. The principal investigator interviewed the patients in the outpatient department of a public hospital. Each interview lasted 10 to 20 minutes. Data were collected on a proforma and included basic demographic information such as age, sex, body mass index (BMI), duration of disease, duration of treatment, place of residency, occupation, education, and economic status, addiction, smoking status, the severity of asthma, and pet exposure. A predesigned Asthma Quality of Life Questionnaire (AQLQ) was used with permission [11-16]. This scale, developed in Canada, assesses the quality of life of asthmatic patients and includes physical and emotional health, subjective health status, and domains of functioning that are important to patients. The data were entered and analyzed by using IBM SPSS Statistics for Windows, Version 19.0 (Armonk, NY: IBM Corp.). The mean and standard deviation was calculated for age, BMI, duration of asthma, duration of treatment, and AQLQ score. The frequency and percentage were calculated for sex, the severity of asthma, residency, quality of life (poor/satisfactory), and other factors (BMI, age, sex, education, economic status, smoking habits, pets at home, and asthma severity). Effect modifiers like age, sex, residency, duration of disease, duration of treatment, and the severity of asthma were controlled through multivariate analysis instead of stratification techniques. Multivariate logistic regression was performed to observe the effect of these variables on the quality of life. The regression coefficient and odds ratio, with a 95% CI, were reported. A P-value ≤ .05 was considered significant.

## Results

**Figure 1:**
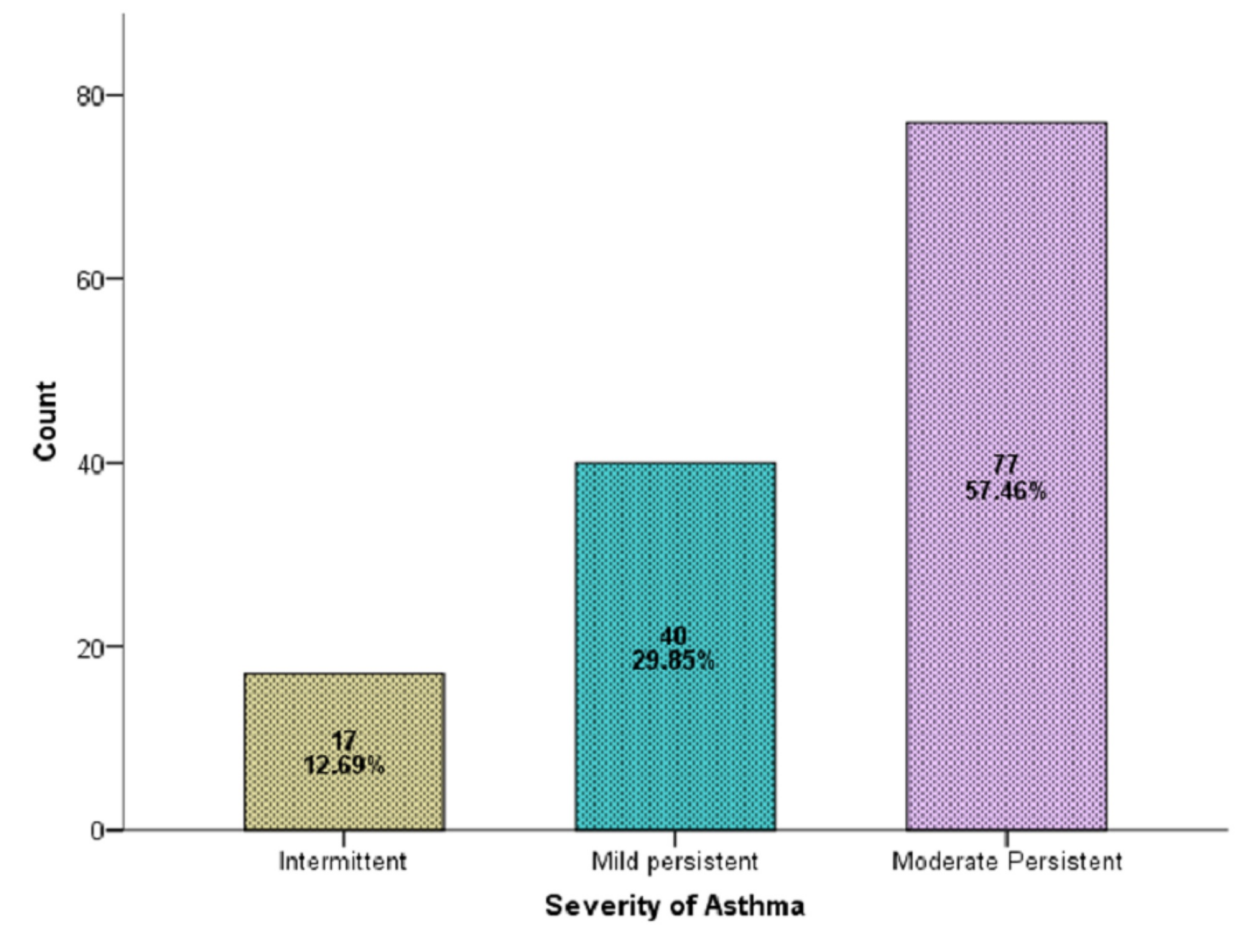
Severity of asthma (n=134)

A total of 134 diagnosed cases of asthma for at least six months and on treatment for at least two weeks were selected in this study. The average age of the patients was 40.6 ± 9.5 years (95% CI: 39.04 to 42.29). Average BMI, duration of asthma, duration of treatment, AQLQ score is presented in Table 1. There were 79 (58.96%) men and 55 (41.04%) women. Intermittent asthma was found in 12.69% of patients, mild asthma in 29.85%, and moderate asthma in 57.46% (Figure 1).

**Table 1:** Descriptive statistics of the characteristics of patients Abbreviations: AQLQ, Asthma Quality of Life Questionnaire; BMI, body mass index

Variables	Mean	95% Confidence Interval for Mean		Standard Deviation
		Upper Bound	Lower Bound	
Age (years)	40.6	39.0	42.2	9.5
BMI (kg/m^2^)	24.38	23.66	25.11	4.19
Duration of asthma (months)	14.50	13.67	15.33	4.87
Duration of treatment (weeks)	6.43	5.88	6.99	3.24
AQLQ Score	3.84	3.52	4.16	1.87

**Figure 2:**
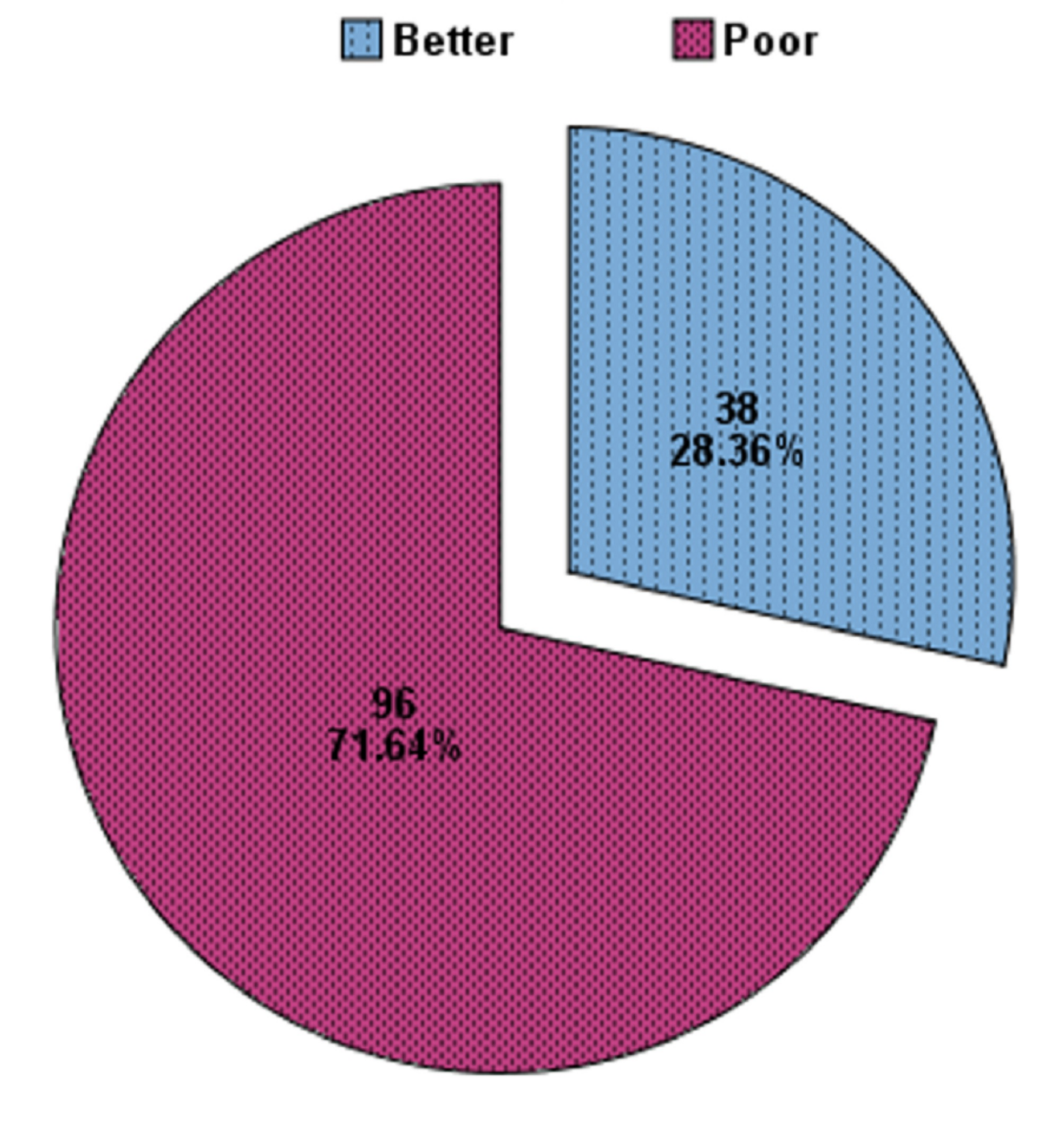
Quality of life in bronchial asthma patients (n=134)

Ninety-six of 134 (71.4%) patients were observed with a poor quality of life in bronchial asthma (Figure 2). Table 2 presents factors contributing to poor quality of life in bronchial asthma patients. An age of 40 years or older, obesity, being female, pets at home, and moderate severity of asthma significantly contributed to poor quality of life while low educational status, family history of asthma, and smoking habits did not have a significant impact. Multivariate logistic regression revealed that advanced age (≥ 40 years), being female, a pet at home, and moderate severity of asthma were four to 13 times more likely to predict a poor quality of life in bronchial asthma (Table 3). The model specification of the logistic regression is also presented in Table 3.

**Table 2:** Factors leading to poor quality of life in bronchial asthma patients Abbreviation: BMI, body mass index

Factors	N	Quality of life		P-value
		Poor	Better	
Advanced age				
≥40 Years	73	61 (83.6%)	12 (16.4%)	.001
<40 Years	61	35 (57.4%)	26 (42.6%)	
Obesity				
BMI ≥ 25 kg/m^2^	52	47 (90.4%)	5 (9.6%)	.0005
BMI < 25 kg/m^2^	82	49 (59.8%)	33 (40.2%)	
Gender				
Men	79	48 (60.8%)	31 (39.2%)	.001
Women	55	48 (87.3%)	7 (12.7%)	
Education Level				
Uneducated	88	67 (76.1%)	21 (23.9%)	.100
Educated	46	29 (63%)	17 (37%)	
Socioeconomic Status				
Low	56	43 (76.8%)	13 (23.2%)	.383
Middle	74	51 (68.9%)	23 (31.1%)	
High	4	2 (50%)	2 (50%)	
Family History of Asthma				
Yes	66	53 (80.3%)	13 (19.7%)	.028
No	68	43 (63.2%)	25 (36.8%)	
Smoker				
Yes	59	46 (78%)	13 (22%)	.150
No	75	50 (66.7%)	25 (33.3%)	
Pets at home				
Yes	42	39 (92.9%)	3 (7.1%)	.0005
No	92	57 (62%)	35 (38%)	
Severity of Asthma				
Moderate	77	25 (43.9%)	32 (56.1%)	.0005
Mild and intermitted	57	71 (92.2%)	6 (7.8%)	

**Table 3:** Multivariate logistic regression model to predict a poor quality of life in bronchial asthma Abbreviations: BMI, body mass index; CI, confidence interval; OR, odds ratio; SE, standard error. Dependent variable = Quality of Life (Poor, Better) Model Summary: Model Accuracy = 90.3%; -2 Log likelihood = 83.14; Cox & Snell R Square = 43.6%; Nagelkerke R Square= 62.5%

Variables	Regression Coefficient	SE	P-value	OR	95% CI for OR	
					Lower	Upper
Advance Age (≥ 40 Years)	1.387	.592	.019	4.00	1.25	12.76
Obesity (BMI ≥25 kg/m^2^)	.844	.671	.208	2.32	0.62	8.65
Female Gender	1.732	.727	.017	5.65	1.36	23.50
Uneducated Patients	.617	.666	.354	1.85	0.50	6.84
Low Socioeconomic status	.638	.683	.350	1.89	0.49	7.21
Family History of Asthma (Yes)	.862	.575	.134	2.36	0.76	7.30
Smoker (Yes)	1.112	.688	.106	3.04	0.79	11.71
Pet At Home(Yes)	1.806	.821	.028	6.08	1.21	30.45
Residency (Rural)	.268	.601	.656	1.30	0.40	4.24
Duration of Asthma (Months)	.087	.080	.278	1.09	0.93	1.27
Duration of Treatment of Asthma (weeks)	-.046	.097	.636	0.95	0.78	1.15
Severity of Asthma (Moderate)	2.617	.672	.0005	13.68	3.66	51.12
Constant	-4.669	1.810	.010	.009		

## Discussion

Our patient population age and male-to-female ratio were similar to the demographics of similar studies reported by Gonzalez-Barcala [10] and Nalina and Chandra [17].

Most of our patients (71.4%) reported a poor quality of life with bronchial asthma, which was higher than the percentage of those reporting a poor quality of life in the report by Motaghi-Nejad et al., who found that only 48.3% of patients reported a poor quality of life [9]. The reasons for the higher incidence of poor quality of life in our patients were likely due to our study population’s more advanced age (> 40 years) and lower educational and socioeconomic status than those in Motaghi-Nejad et al.’s patient population.

In the present study, advanced age ≥ 40 years, obesity, being female, family history of asthma, pets at home, and moderate severity of asthma was significantly contributors to poor quality of life. Gonzalez-Barcala et al. reported similar findings but also found that a low education level (56.5%) and smoking status (23.3%) were associated with a poor HRQoL. These findings were consistent with our results [10]. However, unlike Gonzalez-Barcala et al., we did not address the impact of recurrent admissions on the HRQoL [10].

Even though no association was seen among age and HRQoL [18,19], several authors have noticed a decrease in HRQoL with increasing age [20,21]. Many factors are associated with age and illness, including immunosenescence [22-24]. As comorbidities increase with age, they contribute to the symptomatology and even prohibit the use of certain asthma medications due to contraindications [22,23].

Lower health proficiency has been reported in patients with a lower education level. Likewise, lower numerical aptitudes, progressively postponed determination of asthma, more unfortunate access to social status, or poorer health status could add to the decrease in HRQoL seen in these patients [25,26].

Our study was limited in that the research reflects patients from a single hospital, which means our findings may not be generalizable across a wider geographic population. It is, therefore, recommended that similar studies be conducted in other locations across Pakistan to gain a more accurate assessment of a broad population.

## Conclusions

This study identified several factors responsible for the poor quality of life of patients with asthma. These factors consisted of advanced age, increased asthma severity, poor control of asthma, low education level, and low socioeconomic status. Given the relevant impact of economic and education levels, it is essential that health care providers ensure that patients receive proper education for the prevention of asthma symptoms and provide supportive care when possible to help patients achieve a good quality of life.
